# LBSS: A Lightweight Blockchain-Based Security Scheme for IoT-Enabled Healthcare Environment

**DOI:** 10.3390/s22207948

**Published:** 2022-10-18

**Authors:** Omar Said

**Affiliations:** 1 Department of Information Technology, College of Computers and Information Technology, Taif University, P.O. Box 11099, Taif 21944, Saudi Arabia; o.saeed@tu.edu.sa; 2 Mathematics and Computer Science Department, Faculty of Science, Menoufia University, Shebin Elkom 32511, Egypt

**Keywords:** blockchain, healthcare, IoT, IoT simulation, security, IoT security

## Abstract

Recently, global healthcare has made great progress with the use of Internet of Things technology. However, for there to be excellent patient care, there must be a high degree of safety for the IoT health system. There has been a massive increase in hacking systems and the theft of sensitive and highly confidential information from large health centers and hospitals. That is why establishing a highly secure and reliable healthcare system has become a top priority. In this paper, a security scheme for the IoT-enabled healthcare environment, LBSS, is proposed. This security scheme comprises three security mechanisms. The first mechanism is based on the blockchain technology and is used for transaction integrity. The second mechanism is used to store the healthcare system data in a secure manner through the distribution of its data records among multiple servers. The third mechanism is used to access the healthcare data after applying a proposed authorization test. To minimize the security overhead, the healthcare data is prioritized in regard to its importance. Therefore, each security mechanism has specific steps for each level of data importance. Finally, the NS3 package is used to construct a simulation environment for IoT-enabled healthcare systems to measure the proposed security scheme performance. The simulation results proved that the proposed healthcare security scheme outperformed the traditional models in regard to the performance metrics.

## 1. Introduction

Healthcare can be defined as a large-scale ecosystem that includes many components, such as health insurance, medicine, health facilities, warehouses, robots, sensors, and more. Modern technologies have had the greatest impact on health practices and have transformed them from traditional practices to technological practices, such as monitoring human health using sensors and wearable devices such as smart watches and wristbands. This may be required for a group of patients, such as those with abnormal blood pressure or diabetes, who need periodic monitoring and rapid intervention by specialists to find quick and effective solutions [1,2].

The Internet of Things (IoT) technology connects a group of computers, tablets, mobile phones, and other devices that have a Central Processing Unit (CPU) to handle programs or applications. The main goal of IoT technology is to extend the function of the Internet in order to link the deaf things that are not equipped with CPUs, such as daily live tools, people, medical equipment, and other tracking sensors. Accordingly, there are many applications of IoT technology in various areas of life, such as the military, security, transportation, and economy. All of these fields contribute to the construction of smart cities, which leads to the promised goal of IoT technology, which is a smart world that considers the entire universe as one entity with autonomous management (i.e., without human intervention) [3].

There is a boom in the IoT market which is related to healthcare because of the presence of high-speed Internet. This leads to the creation of the appropriate conditions for healthcare devices to work and operate in one big network as well as for the spread of sensors that support most healthcare devices and equipment. Furthermore, the medical environment is developed technologically and humanely to keep pace with this tremendous development in the information technology and artificial intelligence sectors. Moreover, devices and IoT applications that are gradually adapted to patients and healthcare workers are clearly increasing. Additionally, all components of healthcare, such as patients and doctors, benefit from IoT technology and its applications in monitoring, tracking, maintaining, treating, and more. The IoT-based healthcare system should cover many locations, which leads to a huge number of communicating devices that produce a massive number of gigabytes. The data produced are transmitted through many types of transmission media and processed by different types of users [4].

Sharing data within healthcare systems that are based on IoT technology is an important issue. In these systems, the data is usually shared through cloud-computing technology. Patient data is collected and uploaded to the cloud and is then available to users. Subsequently, it is distributed to authorized persons, such as specialists and administrators, according to the type of data and patients. After that, an initial diagnosis is proposed and then a treatment is prescribed where errors, which may arise because of the manual writing of data, are eliminated. Afterward, data may be sent or received in a real-time manner, if required, through the cloud-computing servers. Because of the presence of sensitive data on the cloud, it may be insecure for several internal or external reasons. For example, the authorized persons to manage the health data centers may be dishonest and tamper with this data. Therefore, there were great security challenges for healthcare systems that depend on the IoT technology, which has unique specifications such as a massive number of terabytes of data, a large number of cloud-computing servers, their extension in many locations, and a large number of users and huge transactions that may have occurred for health data within a short period [5]. The main contributions of this work:

- Apply blockchain technology for data transaction integrity in the IoT-enabled healthcare environment;
- Propose a security mechanism for data storing in the IoT-enabled healthcare environment;
- Propose a security mechanism for data access in the IoT-enabled healthcare environment;
- Construct a simulation for the IoT-enabled healthcare environment and examine the efficiency of the proposed security scheme;
- Finally, show and discuss the simulation results.

The organization of the paper is stated as follows: Section 2 introduces the related works. Section 3 demonstrates the design of the proposed security scheme. Section 4 presents the simulation construction in addition to the discussion of the results. Finally, the paper is concluded in Section 5.

## 2. Related Works

This paper focuses on the security issue for IoT-enabled healthcare environments. The research, which is related to healthcare and blockchain, is evaluated regarding four main factors. The first factor is “IoT Representation (IoT Rep.)”, which evaluates if the related work represented the IoT environment accurately. The second factor is “IoT Suitability (IoT Sut.)”, which evaluates if the related work was suitable for the IoT environment. The third factor is “Security Weakness (SW)”, which evaluates if the related work had security weakness(es). The fourth factor is “Review (Rev.)”, which means that the related work was just a review article. The related works are evaluated and categorized in Table 1.

## 3. Proposed Security Scheme

**Figure 1:**
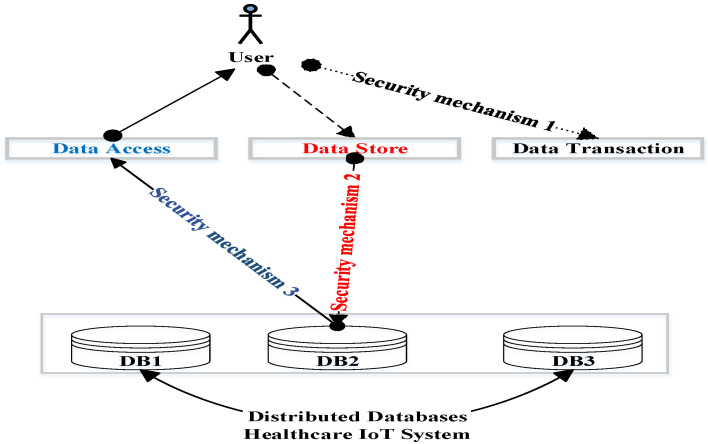
The three mechanisms of the proposed security scheme.

The proposed security scheme provided three security mechanisms: integrity, confidentiality, and availability. The integrity mechanism ensures that the healthcare data transactions are accomplished correctly; see Section 3.1. The confidentiality mechanism guarantees that those authorized to do so can only access sensitive healthcare data. The availability mechanism guarantees that the healthcare data should be available to those who need it; see Figure 1. For confidentiality and availability, see Section 3.2.

One of the challenges of IoT technology is scalability, which causes a huge number of heterogeneous things to suddenly join or leave this environment in addition to a massive number of terabytes of exchanged data within short periods. To face these challenges in the proposed security scheme, the IoT-based healthcare system is divided into many clusters. Each cluster may comprise heterogeneous or homogenous things (devices, tools, and users). The clustering process is achieved based on available network resources, size and type of transmitted data, number of things, and the level of importance of things and data. The clustering process, which is based on constructing a clustering-based attribute selection scale after partitioning the training sample into a number of clusters, is described in [33].

In the sections below, the three mechanisms are discussed in addition to mathematical notations.

### 3.1. Integrity

The healthcare system comprises different types of things. Each thing has a type of exchanged data. Therefore, the healthcare environment has many types of data with different levels of importance. Hence, healthcare data is classified into many levels depending on its importance such that each level can be handled with different procedures [34]. This concept can lead us to the core idea of the proposed integrity factor, and the blockchain technology is used to ensure that each transaction is correctly verified. First, each transaction can be stored as a block, each containing the hash function of the previous block. In case any transaction needs to be added to the chain, it should be verified by a group of persons which are called “miners”. There are many mechanisms to prove the transaction, such as Proof of Work (PoW), Proof of Stake (PoS), and others [35]. As stated previously, the main challenge with the healthcare system based on IoT technology is the terabytes of data (stored, exchanged, and processed). This makes the use of one transaction verification mechanism so difficult (if not impossible). So, in the proposed blockchain mechanism, a mixture of transaction verification mechanisms is applied depending on the type of healthcare data (i.e., importance level). For more clarification, the proposed model classifies the healthcare data into “N” levels of importance such that the first level is assigned to the most important level of data, the second level is assigned to the next level of importance, and so on. This provides flexibility to create other levels of importance, especially for an IoT-based healthcare system. For an accurate determination of the importance of healthcare data, the data type should be combined with the thing’s state. For example, in case of danger, a patient’s status, such as cancer patients, prescriptions, and prescription changes, will have to be completed in an accurate manner and under the supervision of an expert (i.e., in most cases specialists are necessary but insufficient). On the contrary, a patient undergoing a simple general surgery must have accurate anesthesia calculations.

**Figure 2:**
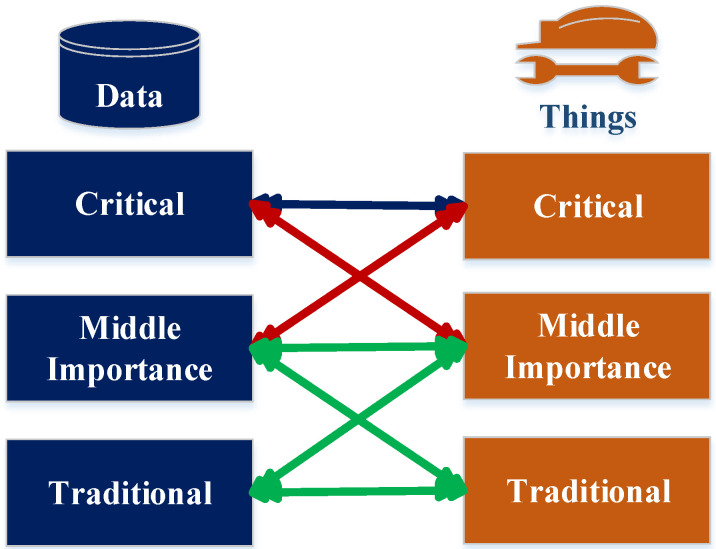
The levels of data importance for data combined with things.

To clarify the model idea, three levels of importance are used and applied for data and things. These three levels of importance are critical, middle, and traditional. The first level of importance is assigned to the data package, which comprises the critical things described by critical data. The second level of importance is assigned to the data package, which comprises the critical things that are described by the data of middle importance in addition to the critical data, which is related to the things of middle importance. The third level of importance is assigned to the data package, which comprises the things of middle importance that are related to the data of middle importance, the data of middle importance that is related to the traditional things, the things of middle importance that are related to the traditional data, and the traditional data that is related the traditional things; see Figure 2.

The importance level of data may be changed by a time period. For example, the timing of blood pressure measurement for a hypertensive patient is sometimes crucial. This is because it can be performed as a routine or considered an important issue to the point where it may save this patient from death. In addition, a certain result from forensic medicine may be used in court and change the direction of judgment, or it may be used only for experiments.

The classification of things and data should be achieved by a high-level committee which is constructed by an elected administration in the healthcare system. The output of the classification process may be changed by a change of committee or over time. Changing the specialists’ committee may affect the outcome of the classification process because each specialist has a point of view about the importance of things and data. For example, there are specialists who consider the data of the medical analysis for a particular disease to be very important, and other specialists may consider this data as traditional. In addition, the time factor is important in changing the result of the classification process, as some data at a certain time can be considered important, while the same data at another time may become unimportant. Therefore, the process of things and data classification may be dynamically changed.

**Figure 3:**
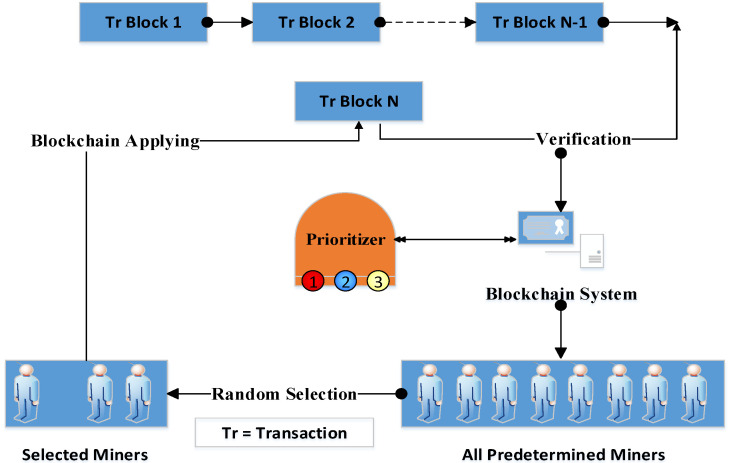
Data priority center, data blocks, and miners’ relationship.

Each level of importance should have a group of miners. These miners can be elected, or it is possible to appoint them according to the protocol or regulations at the hospital. For example, the head of the oncology department, the head of the narcotics department, the director of the hospital, the head of the mental health department, the physician, and the pharmacist in charge of drugs in the hospital may be considered miners who verify the drug transactions. This idea provides scalability, which is one of the challenges of blockchain such that, in case of an extension of healthcare data, the number of miners should be decreased to include the most experienced specialists and high-level managers for critical cases. To differentiate between the old security systems, which verify the transaction through all the available miners, and our proposed scheme, the miners should be selected from different organizations in various locations. This miner selection rule came from IoT envisioning (i.e., the healthcare system that is based on IoT technology transforms many health organizations into one large organization to provide an advantage in selecting miners from different countries, which gives the transaction verification process more credibility and security; see Figure 3). As stated in the bitcoin blockchain system [36], the data is divided into blocks. Each block comprises a group of control fields in addition to the data. One of the most important fields in the data block is the hash function. Each data block has a hash function from the previous block. In the proposed scheme, a hash function is used to condense each transaction.

This function is complex because it consists of a number of simple hash functions. The complexity degree of the hash function depends on the data importance level on which the transaction took place. The number of simple hash functions equals the number of miners who are authorized to check the transactions. Every miner provides the system with its simple hash function in case of their agreement on the transaction.

**Figure 4:**
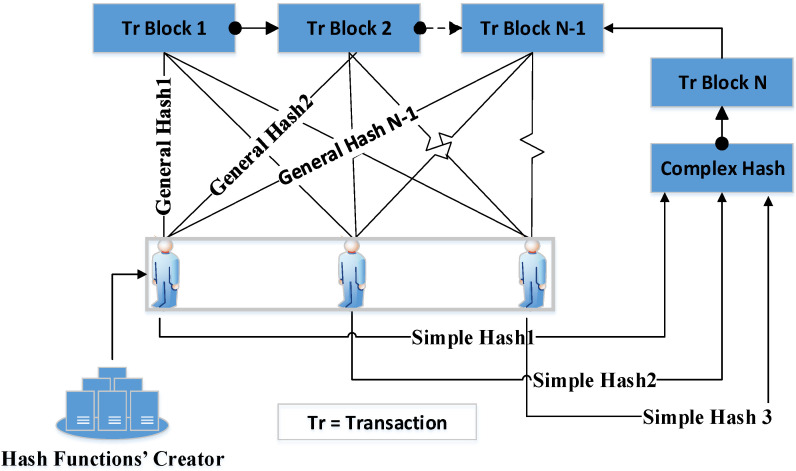
Using simple and complex hash functions.

The completion of the approval by all of the miners means that the simple hash functions are collected to create the complex hash function which, in turn, is used to test whether the transaction is accepted or not. If one miner does not pass the transaction, then their simple hash function is not sent to the security system. Therefore, the complex function will be considered incomplete, which means the transaction is rejected. There is a complex hash function that is assigned to each transaction block such that each miner uses a different simple hash function to test the current transaction and access the old complex hash functions in addition to their old simple hash functions to review the old transactions; see Figure 4. The simple and the complex hash functions are created using the “Hash Function Creator” component. For further description, see Algorithm 1.
** Algorithm 1: Transaction Integrity** D_I_ Data Importance L Importance Level n Number of Importance Levels H_Tr_ Hashed Transaction H_C_ Complex Hash m Number of Minors H_S_ Simple Hash SS Security Server** Algorithm Begin** 1: D_I_ = L[i], 1 < i < n 2: For i = 1 to L  3:                 Begin 4:                 H_Tr_ = H_C_[i](T_r_) 5:                
$H_{C}[i]=\sum_{j=1}^{m}H_{S}\left[j\right]$
 6:                 For j = 1 to m 7:                          
$m_{j}$
$\to H_{S}\left[j\right]$
 8:                 While (T_r_)  9:                          Begin 10:                          $m_{j}\to H_{S}\left[j\right]$ →SS 11:                          $\mathrm{SS}=H_{S}\left[j\right]+H_{S}\left[j+1\right]$ … $H_{S}\left[m\right]$
 12:                         
$\mathrm{IF}\;\mathrm{SS}=\sum_{j=1}^{m}H_{S}\left[j\right]$
 13:                                     
$T_{r}\to Succeed$
 14:                          Else 15:                               
$T_{r}\to Failed$
 16:                          End 17:                   End 18:** End Algorithm**

### 3.2. Confidentiality and Availability

**Figure 5:**
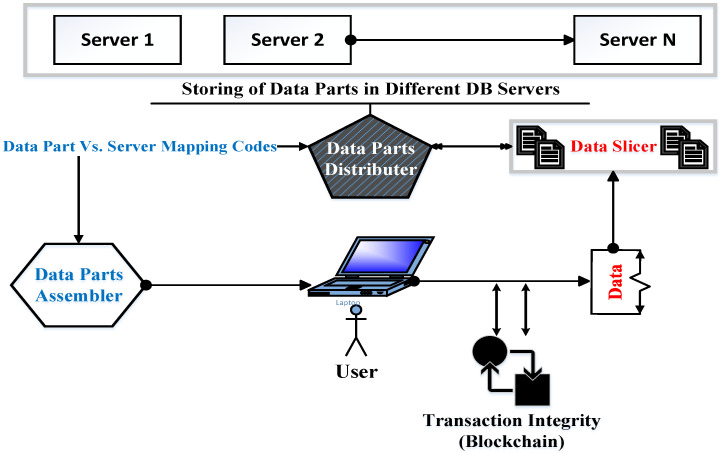
The general view of data distribution over servers.

One of the most important security issues in the healthcare system is the protection of data from unauthorized viewing or accessing. Additionally, the proposed security scheme should guarantee, for authorized users, access to the system’s resources. In traditional healthcare systems, the data that belongs to a healthcare organization is stored on its servers as complete units, which means that everything related to a specific patient is stored as a group of records in one storage database unit. This means any breach in the security system may allow access to patients’ records. To avoid this security weakness, the proposed scheme used the data fragmentation idea. The patient’s data is transformed into a group of records, and each patient record is divided into a group of blocks. These blocks are distributed by the “distributer” component over different servers inside or outside of the organization; see Figure 5.

**Figure 6:**
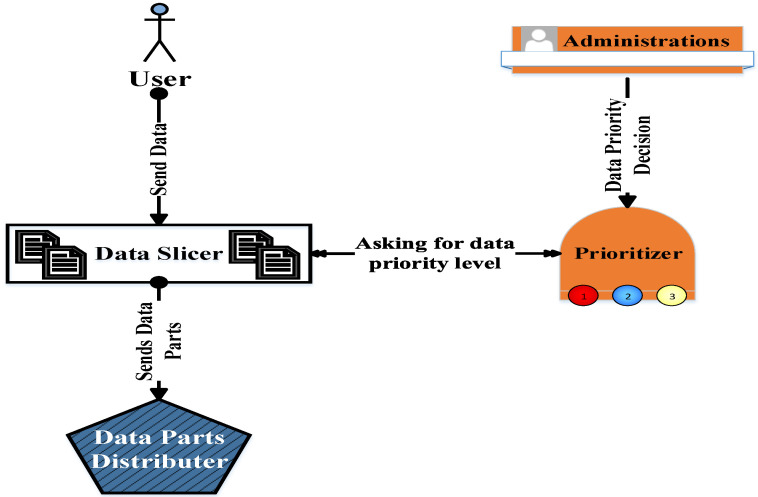
Prioritizer and slicer relationship.

To decrease the overload of the proposed mechanism—in the case of lower-importance data—its records will be directly distributed over the database servers without transformation into blocks. Therefore, the “prioritizer” component should be consulted to determine what will be conducted in the data fragmentation by the “slicer” component; see Figure 6. The function of the “prioritizer” component is achieved using the technique stated in [37] which used the queueing theory by assigning one queue for each importance level. The data in each queue is processed depending on available resources in the entire system.

**Figure 7:**
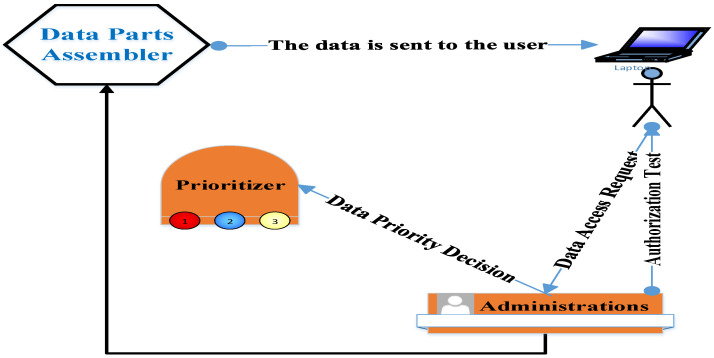
Healthcare data access mechanism.

Each block of data comprises a stamp of its related patient. This stamp may be a picture, a number, or a symbol. The process of choosing the stamp type should take into account that it does not represent an excessive overload on the packet or increase its complexity. This stamp is dynamically exchanged depending on the network status (i.e., available resources). Therefore, the stamp may be first selected as a number. Then, its difficulty is increased by the selection of a more complex form, such as a group of characters. After that, it is transformed into a picture and may be returned to a simple form again. This stamp is used by the “assembler” component to collect the data record after its fragmentation. The “assembler” data is encrypted. The request to access data is sent to the administrator, who, in turn, contacts the “prioritizer” asking about the importance level of the required data. Then, the administrator determines the requirements for this data to be accessed. After that, the administrator contacts the user asking about the required data authorization. Regarding the correct input of the data authorization, the administrator contacts the “assembler” to collect the data and sends it to the intended user; see Figure 7. Communication between the components of the proposed security scheme is achieved using management messages. The structure of these messages is similar to the messages of the Simple Network Management Protocol (SNMP) [38]. Each management message is simple. It comprises the source and destination IP addresses in addition to the required fields such as variables and their values. These management messages are encrypted. In the event of network starvation, the number of management messages will be decreased until the network is returned to its normal status. There are two types of management messages. The first type is called “query” and is used to access specific variables’ values. The second type is called ”response” and is used to send specific data to one or more scheme components.

The authorization data is directly related to its importance level (i.e., the authorization data is changed by adjusting the importance level). The application of this idea in our proposed security scheme can be stated as follows: Each data block (or record) comprises a digital signature of the authorized users. When the authorized user needs to access a healthcare data record, the system should receive the digital signature of that user. To be more accurate, as stated previously, there are several levels of importance for healthcare data. Therefore, each level of importance for the data will have a different security mechanism that should be applied for an access request. In the case of data with a high degree of importance, all users’ electronic signatures must be required to access such data. Hence, the user who sends an access request either enters all of the digital signatures for the users who are authorized to access this type of sensitive data, or this user has a prior authorization deed from those users.

In the same way, electronic signatures will be reduced according to a reduction in the level of importance for the required data. Additionally, each user should determine his/her security behavior, defined by the regular steps that are mostly accomplished within a specified time [39]. This security behavior will be applied as an additional security step for the most important data. So, if the unauthorized user tries to access data and knows the digital signatures of all of the authorized users related to that data, this unauthorized user does not have knowledge of their regular behaviors.

### 3.3. Mathematical Notations

This mathematical notation is based on [40]. Suppose that the number of miners equals “*M*”, where “*L*” is the number of levels of importance. The contents of each data block “*D_C_*” are determined using Equation (1), where “*B^Prev^*” is the previous block, “*H*” is the hash function, “TS” is the timestamp (current time), “b” is the work proof difficulty, and “G” is the target. The “Merkle tree” and “nonce” are omitted from the contents of the block. This is because the time required to test the transaction depends on the predetermined number of miners with simple hashes created by the proposed security scheme. The validity of the transaction “*T_V_*” is determined by Equation (2), where “*P*” is the number of blocks revised by the miners. The time required to add one transaction into the chain “*T_T_*” is determined by Equation (3). The consumption time “*C_T_*” is to ensure all system transactions are determined using Equation (4), where the number of transactions equals “*T*”. The miner is selected from a group of predetermined persons. Additionally, $MN_{L}\left[i\right]$ is the set of miners, and “MNS” is the selected miners. The miners “*M_S_*” are determined using Equation (5).

(1) $$D_{C}=(H(B^{Prev\;\left[a\right]})\;⊕TS(t)\;⊕\;b)\leq G,1<a<P$$

(2) $$T_{V}=\sum_{P=1}^{D}\sum_{j=1}^{M_{i}}H_{j}\;\left(\left(H\left(B^{Prev}\right)⊕TS\left(t\right)⊕b\right)\right),\;0<i<L$$

(3) $$T_{T}=P*\sum_{j=1}^{M_{i}}T_{j}$$

(4) $$C_{T}=\sum_{k=1}^{L}\sum_{j=1}^{T}(P_{k}*\sum_{i=1}^{M}T_{i})$$

(5) $$M_{S}=\frac{P(MN_{L}[i])*P(MN_{L}[i]|MNS)}{P(MN_{L}[i])}$$

## 4. Simulation and Evaluation

The simulation construction process for the healthcare environment, which is based on IoT technology, is demonstrated in the first section. Additionally, the results of the simulation are shown and discussed in the second section.

### 4.1. Simulation Infrastructure

**Figure 8:**
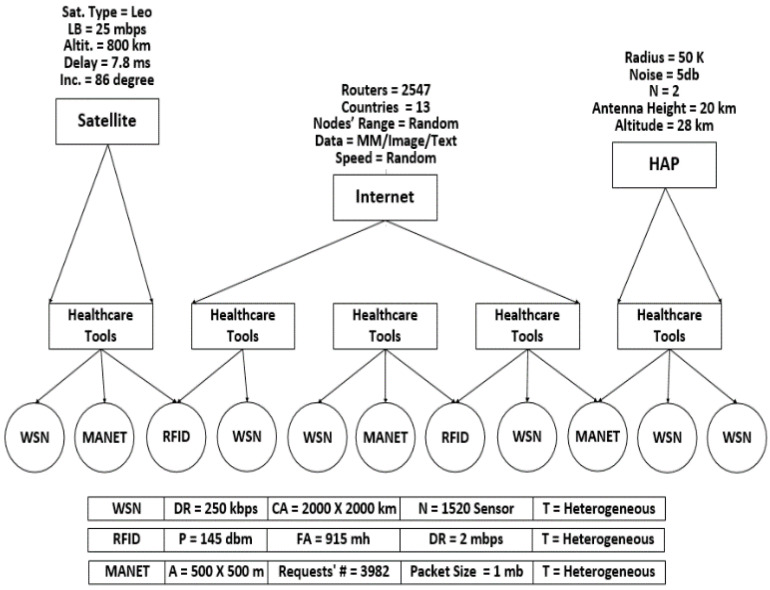
Simulation scenario with networks and coverage tools parameters.

The main challenge with IoT-based healthcare systems is how to represent the nature of the IoT environment. So, the simulation, stated in [41], is the most suitable to reflect the real specification of the IoT environment. This is because it comprises a presentation of three main networks: WSN, Radio-Frequency Identification (RFID), and Mobile Ad hoc Network (MANET). These networks communicate with each other using the Internet, which is also demonstrated. Moreover, this simulation used two alternative coverage methods: satellite and High-Altitude Platform (HAP). These coverage methods are used in case of limitation in the Internet coverage for a group of things. This will provide the healthcare system with representation flexibility through the consideration of the many heterogonous things that are found at many locations with a guarantee that these things will be communicated, even if they are passive things. The simulation scenario, in addition to the parameters’ values for the networks and coverage tools, is stated in Figure 8.

The communication between different packets’ header formats, which are raised due to the usage of different network types, is achieved using the packet transformation idea. Simply, this idea is discussed as follows: encapsulate the received packet with the header that is suitable for the received node. Hence, this node can translate this packet. In the event that the next node has the same type as its previous node, the additional header will remain (i.e., no encapsulation). However, if the next node is different, the header will be de-capsulated and another header will be added in the event that the received node is a new one.

The simulation model, which is used to measure the performance of the proposed security scheme, represented the nature of the IoT environment and is considered the first part of the simulation infrastructure. So, some changes are added to this simulation model to complete the representation of the real nature of healthcare systems, which is considered the second part of the simulation infrastructure. These changes are concluded in Table 2. The selected healthcare devices are the most common ones, such as surgery robotics and glucose monitoring for medical devices, laptops, and barcode readers and monitoring for inventory as well as chemical devices and laptops for pharmacy. Each device can be considered passive or active. The passive things are the things that are not supported by a CPU. However, the active things can connect to the Internet (or any other coverage tool) without additional hardware support. Furthermore, the third part of the simulation infrastructure is related to the representation of security specifications, which are stated in Table 3. The simulation of medical devices is taken from [42,43,44]. Notably, most of the healthcare security parameters are random, ranged values for an accurate representation of the IoT environment.

### 4.2. Results and Discussion

The performance metrics are processing time, the number of miners, average energy consumption, average throughput, average end-to-end delay, packet loss ratio, the access time of healthcare data records, and rate of change between data blocks with different levels of importance. The simulation results of LBSS are compared with the Leila’s model and the Bitcoin model [19,31]. The Bitcoin model was chosen to compare its results with the results of the proposed security scheme because it is one of the most important models that uses the blockchain technology. In addition, the Leila’s model was chosen because it is the closest related work to our proposed security scheme.

**Figure 9:**
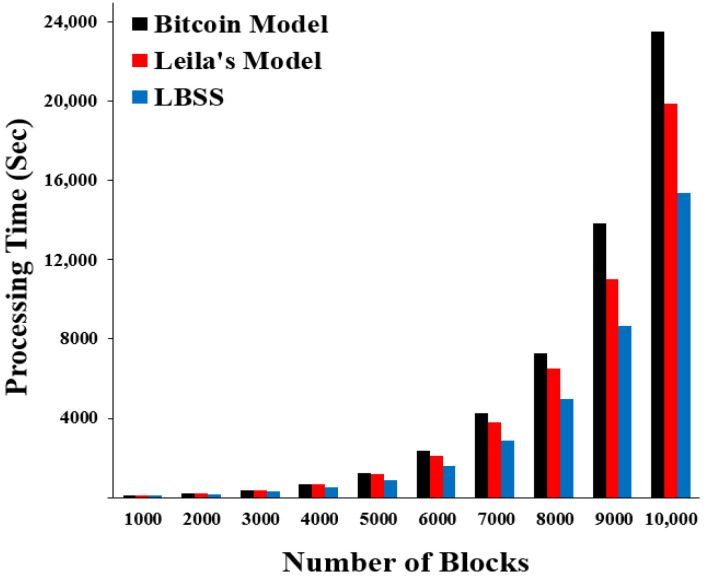
Processing time for transaction integrity.

The processing time performance metric can be measured using a consumption time to validate the transactions, which are represented by blocks in the blockchain system. Figure 9 shows the processing time results for the security scheme, the Leila’s model, and the Bitcoin model. The *x*-axis represents the number of blocks (transactions) and the *y*-axis represents the processing time (seconds). Notably, LBSS has the lowest processing time values. The Leila’s model and the Bitcoin model rank after LBSS. This is explained by the classification of healthcare data into many levels of importance, which decreases the data processing requirements and leads to reduced time consumption.

**Figure 10:**
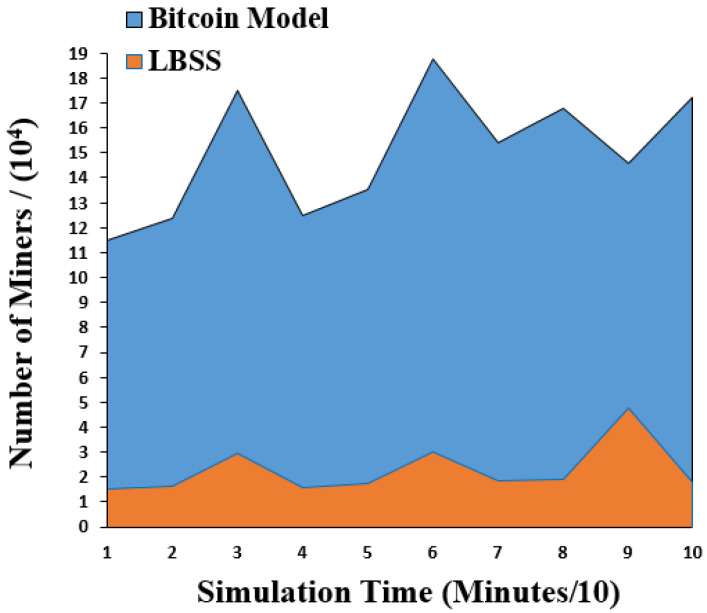
Changing the number of miners over a time.

The number of miners performance metric is measured for LBSS and the Bitcoin model. This is because the number of miners metric for the Leila’s model has the same behavior as the Bitcoin model. This performance metric is measured using the calculation of the average number of miners, which are used to validate the data transactions. Figure 10 shows the result of the changing miner performance metric. The *x*-axis represents the simulation time in minutes divided by ten and the *y*-axis represents the number of miners divided by 10^4^. The number of miners in the proposed security scheme is less than that of the Bitcoin model. This is because the data classification minimizes the number of miners in case there are less important data transactions.

**Figure 11:**
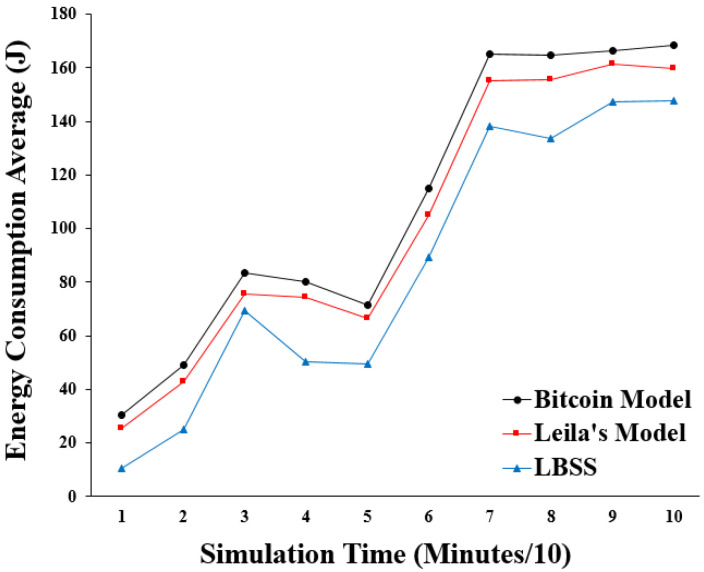
Energy consumption average.

The energy consumption performance metric is measured by the average of the energy consumption values for the energy-based healthcare things in the IoT environment. This performance metric determines the effect of the proposed scheme on the energy-based nodes. Figure 11 shows the results of the energy consumption. The *x*-axis represents the simulation time in minutes divided by ten and the *y*-axis represents the average of the energy consumption in “Joules”. The plot of LBSS has the lowest values for energy consumption within the simulation time. The Leila’s model and the Bitcoin model come after the proposed security scheme, respectively. This is explained by the availability of LBSS to decrease the overhead computations, which lower the energy consumption rates.

**Figure 12:**
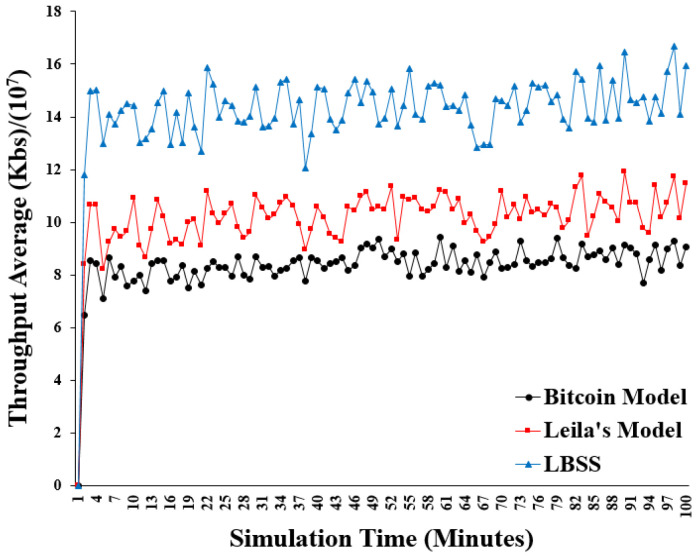
Throughput average.

The throughput performance metric is measured by calculating the size of the data, which is transmitted and received correctly. This performance metric also reflects the effect of the proposed security scheme on the efficiency of the IoT environment because, if the proposed scheme causes a computation overload on the healthcare system, the first metric that will be affected is the size of the correct transmitted data. Figure 12 shows the results of the throughput. The *x*-axis represents the simulation time in minutes and the *y*-axis represents the average throughput in “kb/s.” divided by 10^7^. Notably, the throughput plot of LBSS has the highest values compared with that of the Leila’s model and the Bitcoin model. This is explained by the ability of the proposed security scheme to reduce the number of computations when there is an occurrence of bottlenecks or collisions. Additionally, both the clustering idea and the number of miners affect the throughput performance metric. The clustering idea, which is applied to LBSS and the Leila’s model, reduces their complexity and increases their throughput. However, in LBSS, reducing the number of miners leads to a reduction in the number of transmitted messages, including messages related to the security system. It also leads to a decrease in the negative impact of hash functions’ usage that contributes to an additional increase in the throughput, which was not present in other models. Furthermore, classifying healthcare data into several levels of importance is considered the main factor in determining the behavior of LBSS and making it more flexible.

**Figure 13:**
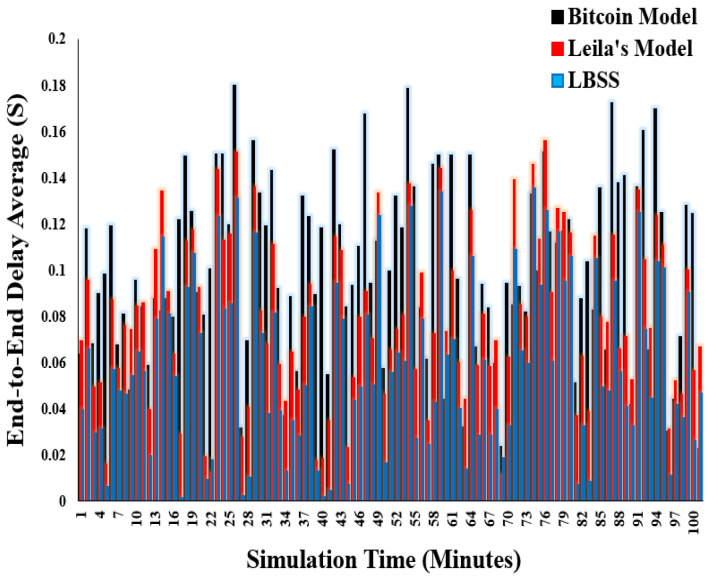
End-to-end delay average.

End-to-end delay is an important performance metric to make sure that the proposed security model does not negatively affect the entire IoT system efficiency. This metric is measured by the calculation of the average of queuing, processing, and transmission delays. Figure 13 shows the end-to-end delay results. The *x*-axis represents the simulation time, and the *y*-axis represents average end-to-end delay. Notably, the end-to-end delay values for the proposed security scheme are less than both the Bitcoin and Leila’s models. In addition, the Bitcoin model has the largest end-to-end delay. This is explained by the flexibility of the proposed scheme to decrease the number of security messages in case of network starvation. However, the complexity of the Bitcoin model is so high that it has negatively affected the IoT-based healthcare environment and increased the average end-to-end delay.

**Figure 14:**
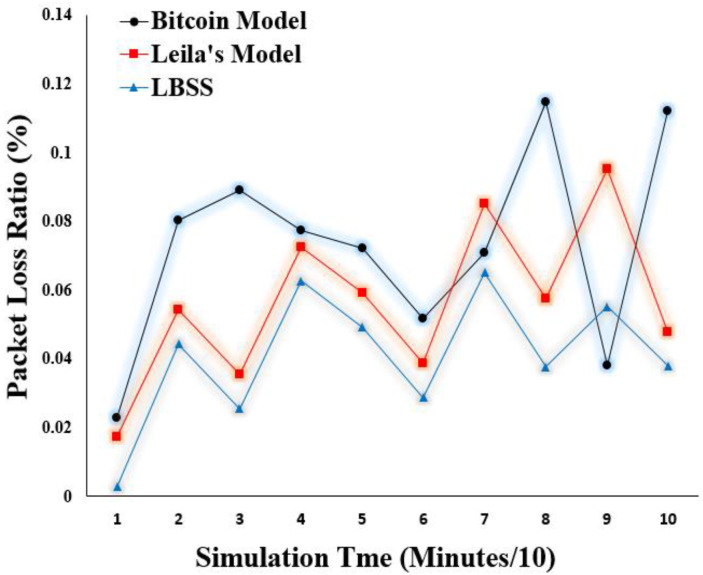
Packet loss ratio.

Packet loss is also a very important performance metric to measure the efficiency of the IoT-based healthcare system. This metric is measured by the ratio of packets that are lost during the transmission process to the total number of sent packets. Figure 14 shows the packet loss ratio results. The *x*-axis represents the simulation time in minutes divided by ten. The *y*-axis represents the packet loss ratio values. Notably, the packet loss ratio for the proposed security scheme is less than that of both the Leila’s and Bitcoin models. This also reflects the efficiency of the proposed security scheme to dynamically change its behavior depending on the network status.

**Figure 15:**
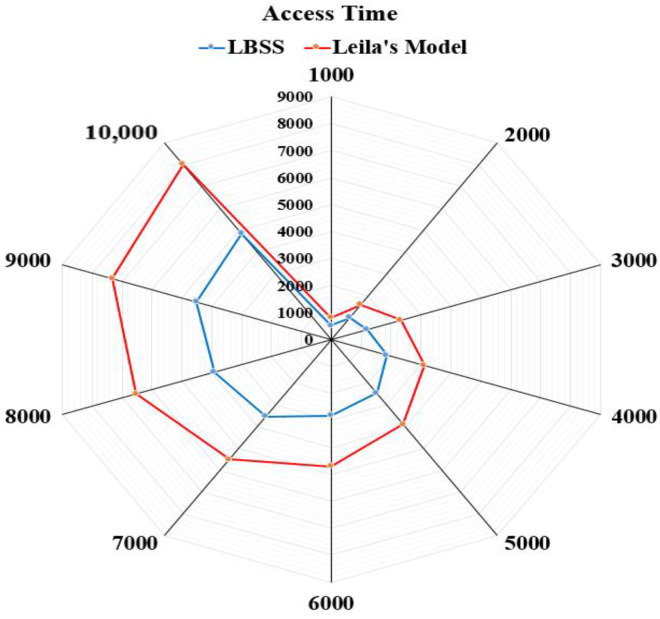
Average consumption time that is required to access healthcare data.

The access time performance metric equals the average consumption time that is required to access healthcare data. This metric measures the performance of the data access mechanism, which is proposed in LBSS. Therefore, it is measured for LBSS and compared to the Leila’s model. Figure 15 shows the results of the access time performance metric. The number of data blocks is ranged from 1000 to 10,000 and the access time is ranged from zero to 9000 “ms”. The access time for the proposed security scheme is less than that of the Leila’s model. This is because the variety of security actions that are executed depends on the level of data importance and type of things.

**Figure 16:**
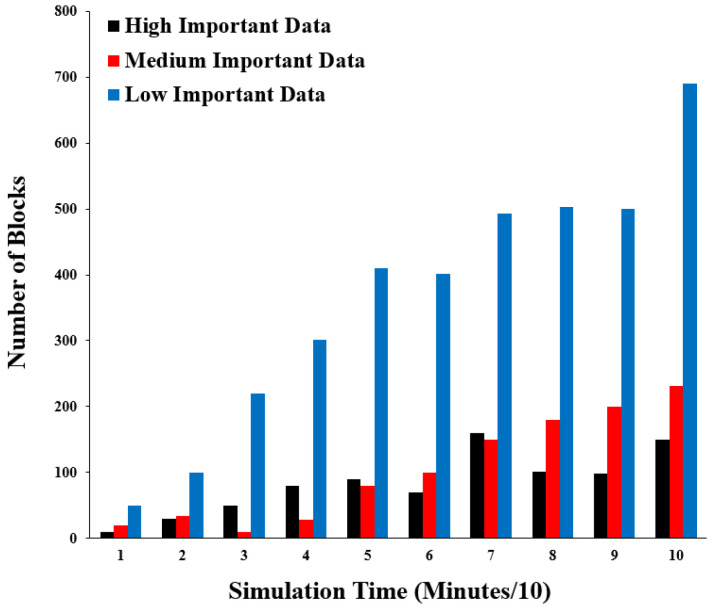
Number of transactions with different levels of importance.

For the transactions with data performance metrics that have different importance levels, they are measured by the number of transactions which occurred for different levels of data importance: high, medium, and low. This is measured to ensure that the changes between the level of data importance work effectively. Figure 16 shows the results of this performance metric. The *x*-axis represents the simulation time in minutes divided by ten and the *y*-axis represents the number of transaction blocks.

The importance levels are distributed between high, medium, and low. Additionally, the number of transaction blocks is mostly arranged in ascending order: low, medium, and high. In the few simulation time points, the high importance level increases the medium importance level, which reflects the real nature of the healthcare environment and comprises occasionally important data.

As shown in Table 4, the simulation results proved that the proposed security scheme, LBSS, outperformed the performance of the related models, the Bitcoin model and the Leila’s model.

## 5. Conclusions

In this paper, a security scheme, LBSS, was proposed for the IoT-enabled healthcare environment. The proposed security scheme deployed blockchain technology to enhance integrity, confidentiality, and availability. Therefore, the healthcare data found in the blockchain was difficult (if not impossible) to access, change, and store without notification and consensus from the all the miners using simple and complex hash functions. Additionally, the proposed scheme was lightweight because its complexity was decreased or increased depending on the data importance level. Moreover, the performance of LBSS was evaluated using an IoT-enabled healthcare simulation environment, and all of its characteristics were precisely generated using the NS3 package. Furthermore, the metrics were chosen to measure the performance of LBSS in addition to evaluating its impact on the entire IoT system. Moreover, the simulation results proved that LBSS outperformed both the Bitcoin model and the Leila’s model. Finally, the recommended areas for future work are deeper analysis of the healthcare data to determine its sensitivity, construction of additional simulation experiments to include other network types such as cellular, applying LBSS to other IoT fields such as the military and agriculture, and comparing LBSS with more complicated systems such as Ethereum.

## Figures and Tables

**Table 1:** Summary of the related works.

Authors	Year	Ref.	Contributions	Limitation Factors			
				IoT Rep.	IoT Sut.	SW	Rev.
Wang et al.	2020	[6]	Proposed a security mechanism for healthcare systems. This mechanism combined the edge cloud and IoT technologies to secure the retrieval of data.	✓	✗	✓	✗
Fotouhi et al.	2020	[7]	Introduced an authentication scheme to prevent attacks targeted at wireless body area networks.	✓	✗	✗	✗
Wang et al.	2020	[8]	Demonstrated a parallel healthcare system in which the blockchain technology and care auditability were applied.	✓	✗	✗	✗
Ismail et al.	2019	[9]	Introduced a blockchain system for healthcare environments. It divided the network into clusters to apply its security model.	✓	✗	✓	✗
Haghparast et al.	2020	[10]	Proposed a security framework for IoT-based e-health systems. This framework consisted of four layers of sensors. Each layer achieved function(s) from the framework target.	✓	✗	✗	✗
Gupta	2017	[11]	Demonstrated a generalized architecture for the security and management of cloud servers in IoT healthcare systems. It divided the cloud centers into three categories to guarantee the secure communication of information.	✓	✗	✗	✗
Ning, et al.	2021	[12]	Demonstrated a blockchain framework for distributed traffic management in transportation systems. It analyzed this framework into two sub-problems to decrease its complexity.	✓	✗	✓	✗
Lakhan et al.	2022	[13]	Identified and ensured the fraud of medical data in addition to privacy preservation at local fog nodes and clouds, with minimum delay and energy consumption.	✓	✗	✓	✗
Manoharan et al.	2022	[14]	Presented a model to analyze the biomedical signals behavior and complete the output tracking mechanism of the transceiver results with low power consumption.	✓	✗	✓	✗
Selvarajan, et al.,	2022	[15]	Introduced a system to minimize the loss of functionalities in the biomedical signals. Additionally, an activation function was presented in the middle stage.	✓	✗	✓	✗
Anitha et al.	2021	[16]	Proposed a security method that detected the replication attack. This security method was for the healthcare system, which was based on a Wireless Sensor Network (WSN).	✗	✓	✓	✗
Benil et al.	2020	[17]	Demonstrated a security scheme to verify and audit medical cloud servers using blockchain technology.	✗	✓	✗	✗
Kong et al. [11]	2019	[18]	Introduced a security model using a neural network and the pre-classification of health data and dynamic gaming theory.	✗	✓	✗	✗
Wang et al. [12]	2020	[19]	Introduced a security framework used to evaluate the security specs of Internet of Health Things (IoHT).	✗	✓	✗	✗
AbouNassar et al.	2020	[20]	Proposed a security model based on blockchain technology. It used a smart contract to enhance the Trustworthy Factor (TF) and establish trustworthy communications in the IoHT infrastructure.	✗	✓	✗	✗
Kavitha et al.	2019	[21]	Introduced a security framework to deal with the security flaws of the personal data records, which were found in healthcare systems. It used the hyperelliptic curve-based public key cryptosystem instead of the traditional cryptographic framework to ensure the group communications were secure.	✗	✓	✗	✗
Tai	2019	[22]	Introduced a security model for IoT healthcare data in which the control operations for the IoT system were adjusted and the user anonymity is considered to introduce an authentication model. Additionally, the control system for IoT has the ability to ensure reliable e-Health services.	✗	✓	✓	✗
Pawar et al.	2018	[23]	Demonstrated a system model that was based on the blockchain technology to manage the health data that were obtained from the medical devices.	✗	✓	✗	✗
Hasanova, et al.	2022	[24]	Presented an algorithm that was based on machine learning technology to predict heart diseases using the blockchain data.	✗	✓	✗	✗
Marwan et al.	2018	[25]	Proposed a security method to prevent unauthorized users from accessing healthcare records. This method used machine-learning technology.	✗	✗	✓	✗
Ding et al.	2019	[26]	Introduced a security system that handles the limited capabilities of sensors. It also verified data integrity and reduced the computation overhead cost.	✗	✗	✓	✗
Li et al.	2020	[27]	Introduced a framework to apply the blockchain technology to guarantee secure data sharing in addition to computing the sensitive data of patients.	✗	✗	✓	✗
Vedaraj et al.	2020	[28]	Provided an effective security framework for the health data in addition to predicting the patients’ diseases. Additionally, it can encrypt and decrypt using a special security algorithm.	✗	✗	✓	✗
Wang et al.	2021	[29]	Introduced a system to quantify the freshness of information using critical-level changes. It was an optimized problem of minimization of the Age-of-Critical-Information (AoCI). Furthermore, an information-aware heuristic algorithm was introduced.	✗	✗	✓	✗
Ning, et al.	2021	[30]	Used Unmanned Aerial Vehicles (UAV) to investigate Multi-access Edge Computing (MEC) that considered edge server deployment and offloading computation. Moreover, two learning algorithms were demonstrated to reach the Nash Equilibrium (NE).	✗	✗	✓	✗
Zarour et al.	2015	[31]	Introduced a study to determine the impact of using blockchain technology on the healthcare system from groups of experts and specialists.	✗	✗	✗	✓
Sharma et al.	2022	[32]	Introduced a literate review of the relationship between the blockchain technology, IoT technology, and the healthcare system.	✗	✗	✗	✓

**Table 2:** Represented healthcare tools in the simulation environment.

Part	Description	Used Network
Patients	Wearable devices (glucometer, fitness bands, heart rate monitoring cuffs, ingestible, and blood pressure), RFID tags, mobiles, and smart watches	WSN and RFID and MANET
Physicians	RFID tags, mobiles, sensors (monitoring and medical), clothes, cars, keys, sonar, surgical tools, laparoscopes, respiratory devices, emergency devices, etc.	WSN and RFID and MANET
Inventories	RFID tags, sensors (weight, temperature, motion, monitoring, and humidity), cargo, lamps, workers, books, offices, etc.	WSN and RFID
Pharmacy	RFID tags, sensors (temperature, touch, motion, monitoring and humidity), mobile applications, refractometer, thermometer, microscope, vacuum oven, etc.	WSN and RFID and MANET
Insurance Company	RFID tags, sensors (temperature, monitoring, and touch), and selected insurance company devices	WSN and RFID

**Table 3:** Security simulation parameters.

Part	Description
Hash	Secure Hash Algorithm (SHA)-256
Block size	1 MB
Number of clusters	Random (10 to 100)
Cluster size	Random (50 to 100)
Total number of miners	Random (10,000 to 200,000)
Number of selected miners	Random (10 to 50)
Number of blocks	Random (144 to 288)
Importance of block	Random (1/2 H: 1/4 M: 1/4 L)
Number of digital signatures	Random (1 to 10)
Total number of nodes	Random (20,000 to 40,000)

**Table 4:** Conclusion of LBSS enhancements compared to both of the Leila’s and Bitcoin models.

Performance Metric	Enhancement
The average processing time for transaction integrity	Decreased by ≈ 22.727% ↓—Leila’s model. Decreased by ≈ 34.349% ↓—Bitcoin model
The changing rate of miners	Decreased by ≈ 84.821% ↓—Bitcoin model.
The energy consumption average	Decreased by ≈ 15.752% ↓—Leila’s model. Decreased by ≈ 21.367% ↓—Bitcoin model.
The average throughput	Increased by ≈ 28.643% ↑—Leila’s model. Increased by ≈ 41.347% ↑—Bitcoin model.
The end-to-end delay	Decreased by ≈ 25.865% ↓—Leila’s model. Decreased by ≈ 42.171% ↓—Bitcoin model.
The packet loss ratio	Decreased by ≈ 27.404% ↓—Leila’s model. Decreased by ≈ 43.880% ↓—Bitcoin model.
The access time for the healthcare data blocks	Decreased by ≈ 39.189% ↓—Leila’s model.

## Data Availability

The data used in this study is created by the simulation package (NS_3_).
